# Chemical Diagnosis of Calcium Pyrophosphate Deposition Disease of the Temporomandibular Joint: A Case Report

**DOI:** 10.3390/diagnostics12030651

**Published:** 2022-03-07

**Authors:** Masahiko Terauchi, Motohiro Uo, Yuki Fukawa, Hiroyuki Yoshitake, Rina Tajima, Tohru Ikeda, Tetsuya Yoda

**Affiliations:** 1 Department of Maxillofacial Surgery, Graduate School of Medical and Dental Sciences, Tokyo Medical and Dental University, 1-5-45 Yushima, Bunkyo, Tokyo 113-8549, Japan; h-yoshitake.mfs@tmd.ac.jp (H.Y.); r_tajima.mfs@tmd.ac.jp (R.T.); yoda.mfs@tmd.ac.jp (T.Y.); 2 Department of Advanced Biomaterials, Graduate School of Medical and Dental Sciences, Tokyo Medical and Dental University, 1-5-45 Yushima, Bunkyo, Tokyo 113-8549, Japan; uo.abm@tmd.ac.jp; 3 Department of Oral Pathology, Graduate School of Medical and Dental Sciences, Tokyo Medical and Dental University, 1-5-45 Yushima, Bunkyo, Tokyo 113-8549, Japan; yfkwmpa@tmd.ac.jp (Y.F.); tohrupth.mpa@tmd.ac.jp (T.I.)

**Keywords:** calcium pyrophosphate dihydrate deposition disease, pseudogout, temporomandibular joint, X-ray diffraction, inductively coupled plasma atomic emission spectroscopy

## Abstract

Calcium pyrophosphate dihydrate (CPPD) deposition disease is a benign disorder characterized by acute gouty arthritis-like attacks and first reported by McCarty. CPPD deposition disease rarely occurs in the temporomandibular joint (TMJ), and although confirmation of positive birefringence by polarized light microscopy is important for diagnosis, it is not reliable because other crystals also show birefringence. We reported a case of CPPD deposition disease of the TMJ that was diagnosed by chemical analysis. A 47-year-old man with a chief complaint of persistent pain in the right TMJ and trismus was referred to our department in 2020. Radiographic examination revealed destruction of the head of the mandibular condyle and cranial base with a neoplastic lesion involving calcification tissue. We suspected CPPD deposition disease and performed enucleation of the white, chalky masses. Histopathologically, we confirmed crystal deposition with weak birefringence. SEM/EDS revealed that the light emitting parts of Ca and P corresponded with the bright part of the SEM image. Through X-ray diffraction, almost all peaks were confirmed to be CPPD-derived. Inductively coupled plasma atomic emission spectroscopy revealed a Ca/P ratio of nearly 1. These chemical analyses further support the histological diagnosis of CPPD deposition disease.

## 1. Introduction

McCarty was the first to report a case of calcium pyrophosphate dihydrate (CPPD) crystal deposition disease, a rare benign crystalline arthropathy also known as pseudogout [1,2]. This disease is characterized by the accumulation of CPPD crystals in various intra-articular and periarticular tissues [3]. Unfortunately, its etiology is unknown, but the disease has been associated with metabolic disorders such as hyperparathyroidism, hypothyroidism, hypomagnesemia, and hyperphosphatemia [4,5,6]. Diabetes mellitus is associated with a greater incidence of CPPD deposition disease [1,7]. CPPD deposition disease predominantly involves relatively large joints such as the knee, shoulder, hip, wrist, and pubic symphysis; small joints such as the temporomandibular joint (TMJ) are rarely affected [4,8,9]. Pritzker et al. were the first to describe pseudogout in the TMJ in 1976 [10]. Almost all previously reported cases of CPPD deposition disease of the TMJ were diagnosed using a polarized microscope to find positive birefringence. However, we consider this modality insufficient for diagnosis because, in addition to those in CPPD and gout, many birefringent crystals such as those of calcium oxalate, synthetic steroids, and ethylenediaminetetraacetic acid are present in the joint fluid, joint tissue, and bone [11]. Herein, we describe a case of CPPD deposition disease of the TMJ diagnosed using chemical analyses, scanning electronic microscopy (SEM)/energy-dispersive X-ray spectroscopy (EDS), XRD, and inductively coupled plasma atomic emission spectroscopy (ICP-AES).

## 2. Case Presentation

### 2.1. Clinical Summary

A 47-year-old man with a chief complaint of persistent pain in the right TMJ and trismus was referred to our department in 2020. He experienced a traffic accident approximately 25 years ago, which damaged his liver and pancreas and caused wrist and left shoulder bone fractures. His clinical history appeared related to the accident, in which he had also bruised his right TMJ but had not sought treatment for it. Since the accident, the patient experienced discomfort with irregular sudden pain in the right TMJ. This pain resolved after the use of analgesics at every episode. He visited a local hospital when the frequency and intensity of pain increased in 2020. He was then referred to our department when surgical management was anticipated.

Clinical examination revealed bilateral symmetry of the face. His mouth opening was limited, and there was limited lateral excursion to the left. The maximal mouth opening was 28 mm and accompanied by pain in the right TMJ. His uric acid level was normal.

**Figure 1:**
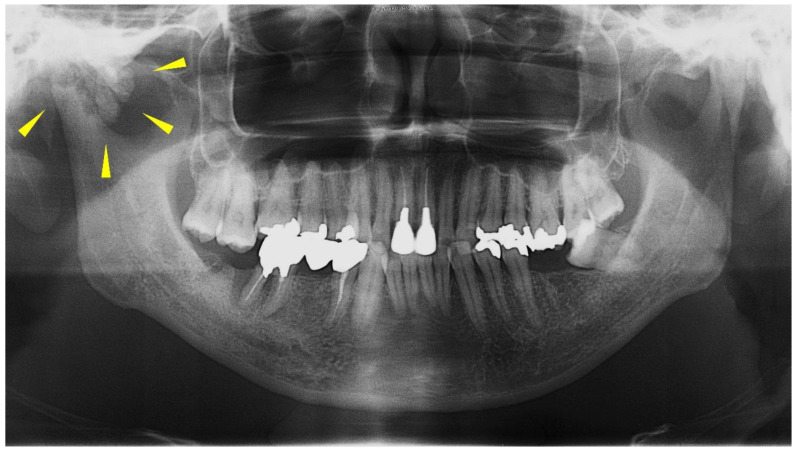
Panoramic radiograph from the first visit. The ill-defined calcification around the mandibular condyle is shown (yellow arrows).

**Figure 2:**
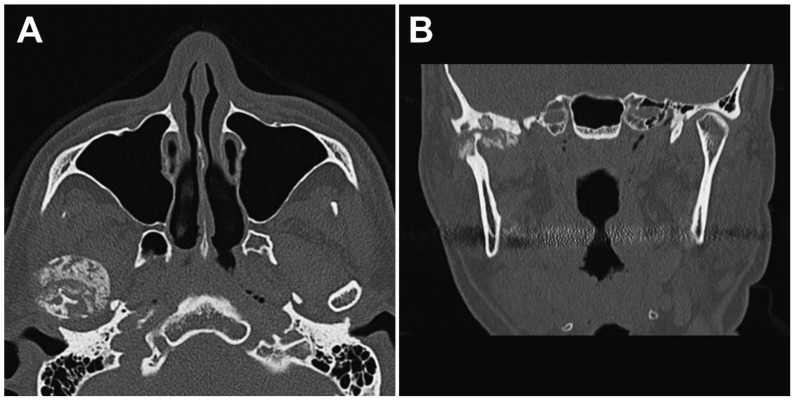
Preoperative computed tomography (CT) images. (**A**) Axial CT images showing the intra-articular localized, non-corticated, and ill-marginated calcified lesion that abuts the articular surface of the glenoid fossa around the right mandibular condyle. (**B**) Coronal CT images of the right temporomandibular joint revealed resorption of part of the mandibular condyle and cranial base.

**Figure 3:**
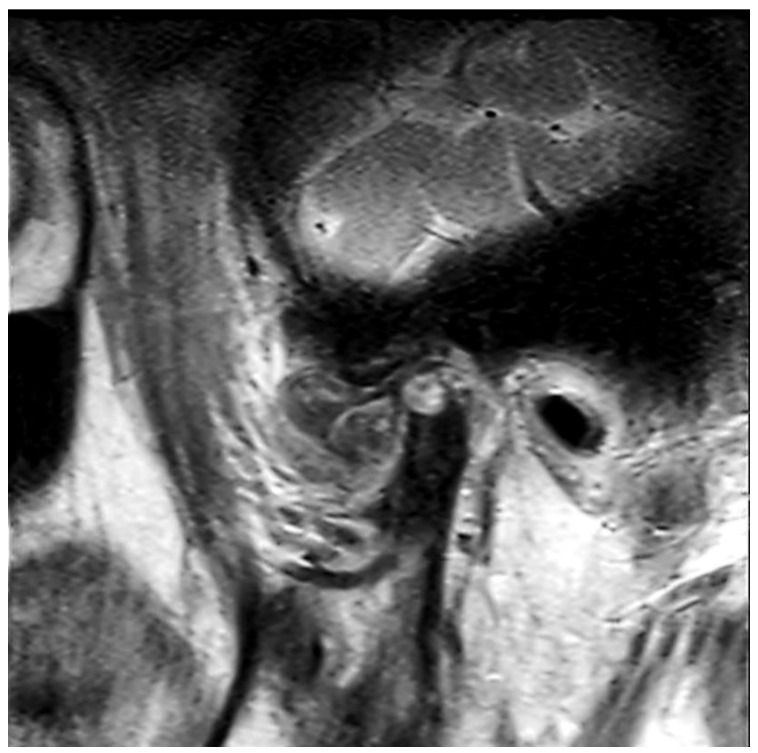
Magnetic resonance image. Proton density-weighted image of the sagittal plane.

**Figure 4:**
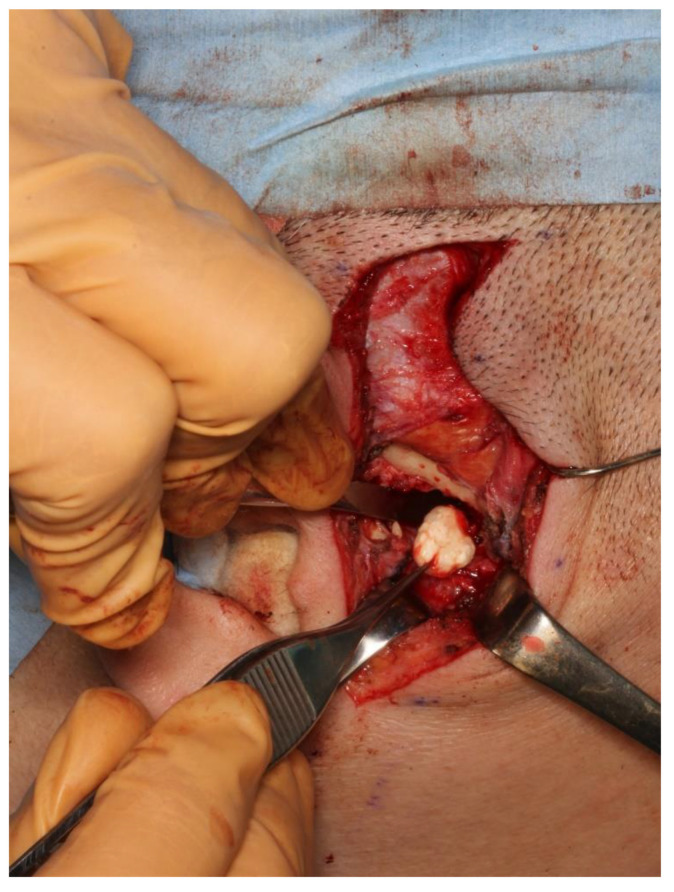
Intraoperative photograph. The whitish calcified tumorous mass was enucleated from the right infratemporal fossa.

**Figure 5:**
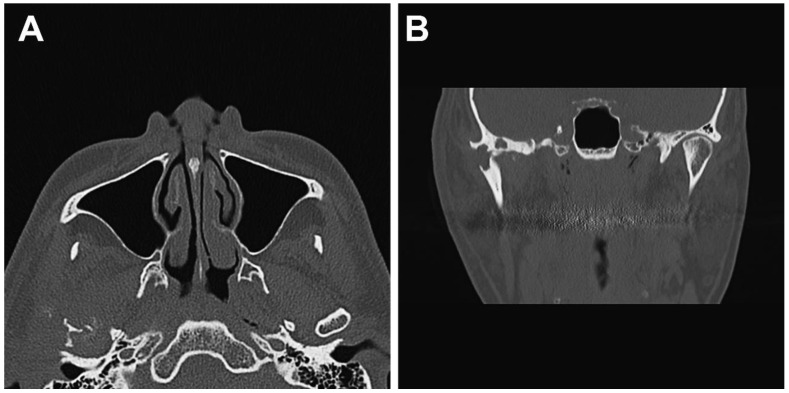
Postoperative computed tomography (CT) images. (**A**) Axial CT images and (**B**) Coronal CT images revealed that the masses were extracted from the right temporomandibular joint.

The panoramic radiograph showed an unclear right mandibular condyle with a cloud-like mass (Figure 1). Computed tomography (CT) revealed that the right mandibular condyle was destroyed, and that mottled-like hard tissues had formed around the condyle as viewed on the axial plane (Figure 2A). Similarly, it was confirmed on the coronal plane that the mandibular fossa and cranial base were destroyed. Furthermore, calcified opacity was observed in the bone resorption fossa (Figure 2B). Proton density-weighted imaging showed no disc dislocation in the right TMJ, and the area corresponding to the upper and lower joint space was filled with uneven hypointensity, and the joint space appeared dilated. Additionally, the high signal inside and granular low-signal images were scattered inside the mandibular condyle and fossa (Figure 3). The left TMJ showed no abnormal findings. Based on these findings, we suspected CPPD deposition disease as a clinical diagnosis and excised the lesion under general anesthesia. The right TMJ was exposed using a preauricular approach. During surgery, we confirmed and removed the white chalk-like masses (Figure 4). These masses were present in the articular capsule, articular eminence, mandibular condyle, the upper and lower joint cavities, and articular disc. The maximum size of the masses was 16 × 5 × 5 mm, although various sizes were extracted. CT images were obtained after surgery, and we confirmed that the masses were extracted from the right temporomandibular joint (Figure 5). The postoperative healing was uneventful. This was six months post-surgery, and although the pain in the right TMJ was persistent when opening the mouth, the maximal mouth opening had improved to 42 mm.

### 2.2. Pathological Findings

**Figure 6:**
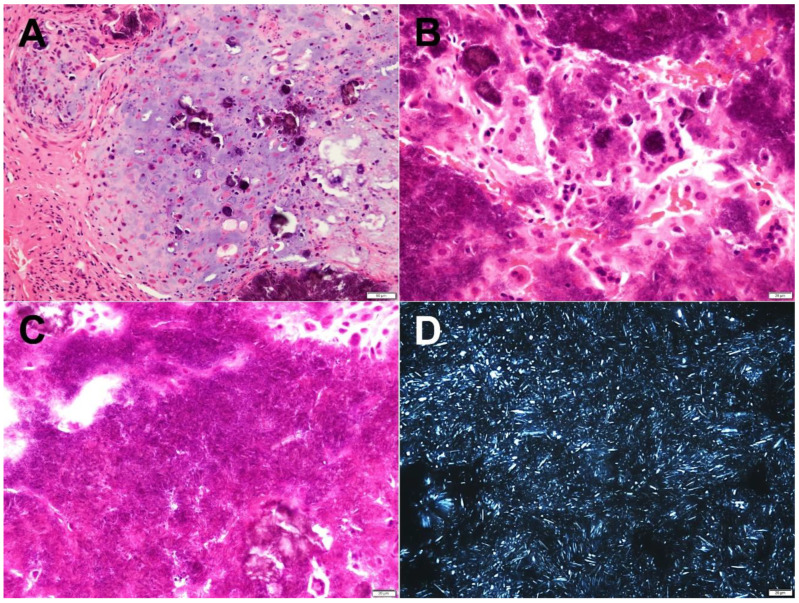
Histopathological examination. Representative specimen from the upper joint cavity showed the histopathological features of calcium pyrophosphate dihydrate deposition disease. (**A**) Chondroid metaplasia forms around basophilic islands of crystalline deposits. (**B**) A foreign body granulomatous reaction with multinucleated giant cells phagocytosing the crystals. (**C**) Deposited crystals appeared rhombus or needle shaped. (**D**) Under polarized light, these crystals demonstrated weak birefringence.

Histologically, the masses consisted of chondroid tissue with island-like or nodular deposition of basophilic crystals (Figure 6A). A foreign body granulomatous reaction was observed in some areas around the crystal deposition (Figure 6B). The crystals appeared rhombus or needle shaped and showed weak birefringence under the polarized light microscopy (Figure 6C,D).

### 2.3. Elemental Analysis Using SEM/EDS

**Figure 7:**
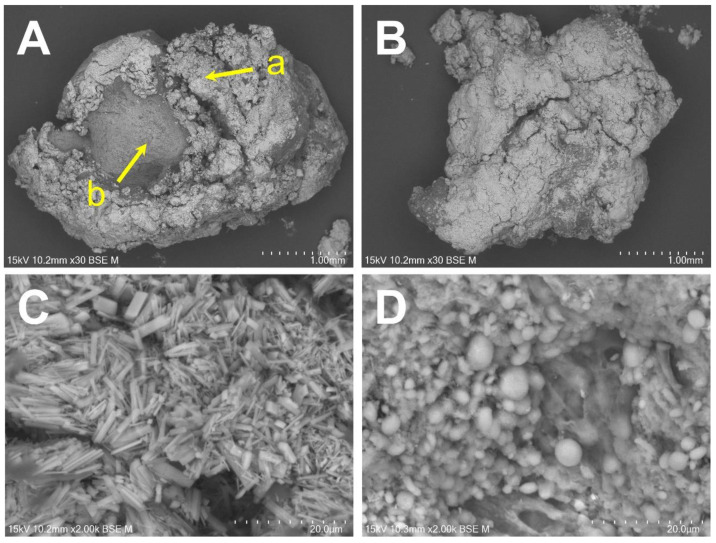
Scanning electronic microscopic images of the masses from the joint cavities. Masses were extracted from the (**A**) upper and (**B**) lower joint cavities (30×). (**C**,**D**) present the 2000× high power fields of (**A-a**) and (**A-b**), respectively.

**Figure 8:**
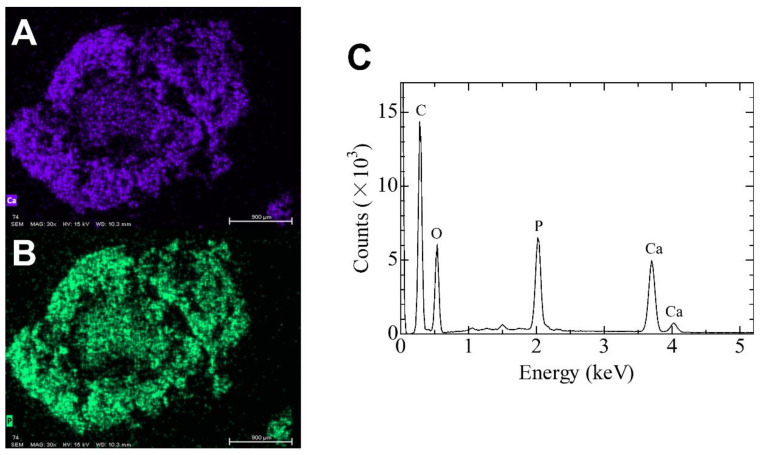
Elemental distribution images of (**A**) Ca and (**B**) P. (**C**) The EDS spectrum for the entire specimen (from Figure 6A) obtained by SEM/EDS.

The two large deposits extracted from the upper and lower joint cavities were chosen as representative specimens for the chemical analysis. SEM/EDS microanalysis was performed to evaluate the calcified mass. Each deposit was fixed with 10% paraformaldehyde solution and washed with distilled water. Thereafter, it was dehydrated in a series of alcohol baths of increasing concentration and dried using vacuum drying. SEM was performed to observe the fine structure around the deposit surface. A carbon coat was formed on these surfaces and observed using SEM (TM4000Plus, Hitachi High-Tech Corporation, Tokyo, Japan) at an acceleration voltage of 15 kV. The elemental distribution around the interface was estimated using EDS (Quantax75 (Oxford Instruments, Oxford, England). The elemental distribution images of the interface were acquired with a resolution of 256 × 200 pixels with an integration time of 200 μs per point. The results are shown in Figure 7. The calcified mass from the upper joint cavity consisted of needle-like crystals, rhomboid masses, and soft tissue that lacked the crystal. However, the specimens from the lower joint cavity consisted of needle-like crystals. Both crystals were the same size with no more than 1 μm thickness and a length of approximately 10 μm. The elemental distribution images and spectrum are shown in Figure 8. The same specimen used in Figure 6A (upper joint cavity) was analyzed. The light emitting parts of Ca and P corresponded with each other. Figure 8C shows the elemental distribution diagram: Ca, P, O, and C were detected. The specimen in Figure 7B (lower joint cavity) was also analyzed, and the same results were obtained (data not shown).

### 2.4. Crystal Phase Analysis Using XRD

The calcified specimens extracted from the upper and lower joint cavities (Figure 6A,B) were washed several times with distilled water, dried at 180 °C for 1 h, and ground into powder using an agate mortar. The crystal phases of the powder specimens were analyzed using XRD (Miniflex, Rigaku Cooporation, Tokyo, Japan) under the following conditions: 40 kV, 15 mA, and 2°/min.

**Figure 9:**
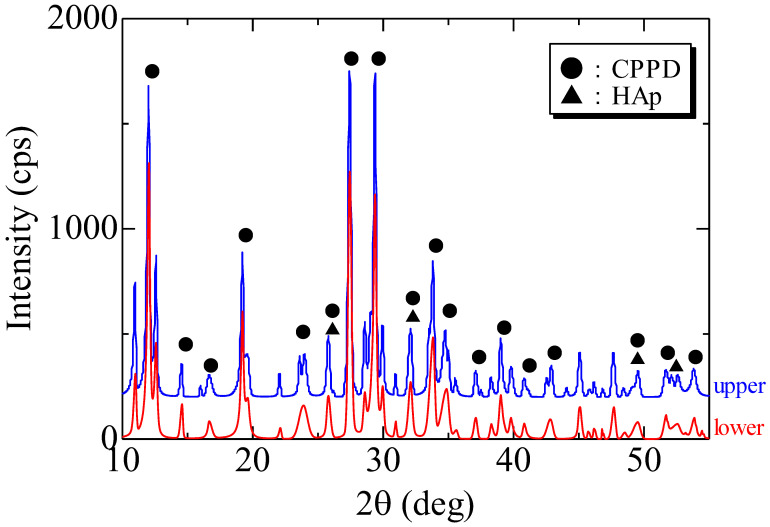
X-ray diffraction. The blue and red wavelengths represent the specimens extracted from the upper and lower joint cavities, respectively. The circles and triangles indicate the intrinsic peaks of calcium pyrophosphate dihydrate (CPPD) and hydroxyapatite (HAp), respectively.

Most diffraction peaks of both crystals were assigned to those of CPPD, and a few small peaks were assigned to those of hydroxyapatite (HAp). Therefore, the main crystal was CPPD (Figure 9).

### 2.5. Quantitative Elemental Analysis for ICP-AES

The tissue concentrations of Ca and P were quantitatively evaluated using ICP-AES. The specimens of the deposits were washed several times with distilled water and weighed while wet (upper: 0.0322 g, lower: 0.0582 g). The specimens were then dissolved in concentrated nitric acid (HNO_3_; 38 *w*/*v*%, UltraPur100, Kanto Chemical Co. Ltd., Tokyo, Japan) overnight at 90 °C. The trace element concentrations in the solutions were quantitated using ICP-AES (Spectro Arcos, Hitachi High-technologies, Tokyo, Japan). Multi-element (100 ppm, XSTC-22, Seishin Trading Co. Ltd., Kobe, Japan) and Sr standard solutions (1000 ppm, Nacalai Tesque, Kyoto, Japan) were used for ICP-AES analyses. The measurement results are presented in Table 1. In the upper cavity specimen, 11.20 wt% Ca and 9.20 wt% P were detected. In the lower cavity specimen, 9.12 wt% Ca and 6.75 wt% P were found (Table 1). Fe, K, Mg, Na, Zn, and Sr were also detected as the trace elements present in the specimens, while the other elements could not be detected or the detection limit or less by this method. In other words, it was clearly composed of elements of biological origin. Accordingly, a Ca/P molar ratio of 0.94 and 1.04 was obtained in the upper and lower cavity specimens, respectively. CPPD is a calcium phosphate that has a Ca/P molar ratio of 1.0. Therefore, the elemental analyses with ICP-AES further supported the histological diagnosis of CPPD deposition disease.

## 3. Discussion

McCarty’s diagnostic criteria for CPPD deposition disease are based on the following: (1) the validation of the specimen by reliable methods such as XRD or chemical analysis or (2) the presence of typical calcific deposition and the detection of crystals suggestive of calcium pyrophosphate deposition through a polarized microscope [1]. The crystal deposits in CPPD deposition disease had a rhomboid structure and were positively birefringent under polarized light, whereas those in gout exhibited negative birefringence. Therefore, birefringence is an important differential diagnostic criterion for gout and CPPD [3,12]. In our case, these crystals clearly demonstrated a rhomboid and rod-shaped appearance, and they exhibited birefringence under a polarized microscope (Figure 6D). Based on these findings, CPPD deposition disease was suspected. However, definitive diagnosis of CPPD can be difficult because not only are these crystals small and often show weak birefringence, but there are also many other birefringent crystals such as those of calcium oxalate, synthetic steroids, and ethylenediaminetetraacetic acid, present in the joint fluid, joint tissue, and bone [11,13]. Therefore, because other quantitative and chemical analyses are required for definitive diagnosis of CPPD deposition disease, we performed SEM/EDS, XRD, and ICP-AES.

Asghar et al. described how crystals demonstrate peaks corresponding to Ca and P in SEM/EDS; therefore SEM/EDS is a rapid and effective method for diagnosing CPPD [3]. In elemental analysis using EDS, only Ca, P, and O derived from CPPD and C and O derived from soft tissue were observed, and the distribution of Ca and P was the same as the bright part of the SEM image (Figure 7A and Figure 8). These results suggest that the specimens contained CPPD. Most previous reports of CPPD deposition disease describe the detection of Ca and P using SEM/EDS or the diagnosis of CPPD based on a Ca/P ratio of approximately 1 on a rough composition analysis using EDS [3,4,5]. However, these diagnostic methods are considered inappropriate for the following reasons: (1) Since there are innumerable calcium phosphate compounds such as HAp, tricalcium phosphate (TCP), octacalcium phosphate, and dibasic calcium phosphate anhydrous, it is not possible to determine the exact calcium phosphate compound present despite the detection of Ca and P (Table 2), so accurate Ca and P concentrations should be determined to distinguish calcium phosphate compounds; and (2) most EDS composition analyses have a “standardless method,” and their accuracy is lower than that of other analyses calibrated with the concentration standard specimens. Therefore, additional analyses are required to definitively diagnose the precipitation as CPPD.

XRD is a powerful method for the crystal phase and structure analyses of inorganic compounds. The basic method for the crystal identification of inorganic compounds through a database is XRD, and if the results are combined with the identification of major elements using EDS elemental analysis, the elements can be identified with high reliability [14]. XRD revealed that all diffraction peaks were consistent with those of CPPD. Even small peaks were thought to be derived from hydroxyapatite, and the main crystals were strongly considered to be derived from CPPD (Figure 9). XRD can help distinguish crystal phase identification and form, but cannot correctly quantify the chemical composition. This method uses a “standardless method,” but SEM/EDS can be used for pseudo-analysis. Thus, the accuracy of the numerical value is questionable.

In this study, we focused on ICP-AES analysis to further accumulate evidence. Bones and teeth are not purely composed of calcium phosphate and often contain divalent cations of Mg, Sr, and Zn instead of Ca (for example, Sr exists at a concentration of one hundred to several hundred parts per million) [15]. Additionally, ICP-AES can help reliably quantify the Ca/P ratio and confirm CPPD based on the chemical composition of the specimen. In CPPD, the Ca/P ratio was 1, which was lower than that of HAp and TCP (Table 1). In our results, the Ca/P ratio in the upper and lower joint cavities was 0.94 and 1.04, respectively. The analysis value retention Ca/P ratio obtained through ICP-AES was approximately 1. These results indicate that there is no possibility that other calcium phosphate compounds are present, which supports the diagnosis of CPPD deposition from the perspective of the chemical composition. In addition, only cations contained in the human organism were detected in our case. In other words, heavy metals and other substances are unlikely to accumulate or be the cause of the problem. Assuming that all the aforementioned Ca values were associated with CPPD deposits, the weight ratio of CPPD in the tissue was estimated to be 40.6 wt% on the upper side and 33.0 wt% on the lower side. Considering this number as wet weight, most of the tissue was CPPD, which corresponds reasonably well with the SEM observations. Thus, we diagnosed CPPD deposition disease of the right TMJ. The diagnosis of CPPD deposition disease by chemical analysis is not simple considering the special equipment and the number of specimens required for analysis. For this reason, in this study, we preoperatively suspected CPPD, consulted with pathologists and engineers, and used chemical analysis for postoperative diagnosis. Collaborating with pathologists and engineers on preoperatively suspected CPPD deposition disease was effective in obtaining a more reliable diagnosis.

## 4. Conclusions

In summary, the diagnosis of CPPD deposition disease of the TMJ is based on the presence of rhomboid positively birefringent crystals; however, because it is considered as a weak diagnostic criterion, performing chemical analyses such as SEM/EDS, XRD, and ICP-AES offers a reliable method for the diagnosis of CPPD deposition disease.

## Figures and Tables

**Table 1:** ICP-AES for quantitative analysis of elements.

Element	Quantity		Unit
	Upper	Lower	
Ca	11.2	9.12	wt%
P	9.2	6.75	
Fe	24	22	μg/g (ppm)
K	153	102	
Mg	274	267	
Na	1920	2140	
Zn	7	16	
Sr	16	12	

**Table 2:** A list of the major calcium phosphate compounds.

Composition Formula	Ca/P Ratio	Name	Abbreviation (Mineral Name)
Ca (H_2_PO_4_)_2_·H_2_O	0.5	Calcium bis(dihydrogenphosphate) monohydrate	MCPM
CaHPO_4_	1	Calcium monohydrogen phosphate	DCPA (monetite)
CaHPO_4_·2H_2_O	1	Calcium hydrogen phosphate dihydrate	DCPD (brushite)
Ca_2_P_2_O_7_	1	Calcium pyrophosphate	
Ca_2_P_2_O_7_·2H_2_O	1	Calcium pyrophosphate dihydrate	CPPD
Ca_8_(PO_4_)_4_(HPO_4_)_2_(OH)_2_	1.33	Octacalcium phosphate	OCP
Ca_3_(PO_4_)_2_	1.5	Tricalcium phosphate	TCP
Ca_10_(PO_4_)_6_(OH)_2_	1.66	Hydroxyapatite	HAp
Ca_4_(PO_4_)_2_O	2	Tetracalcium phosphate	TTCP

## Data Availability

Not applicable.
